# Highly Variable Expression of Merozoite Surface Protein MSPDBL2 in Diverse Plasmodium falciparum Clinical Isolates and Transcriptome Scans for Correlating Genes

**DOI:** 10.1128/mbio.01948-22

**Published:** 2022-08-11

**Authors:** Suzanne E. Hocking, Lindsay B. Stewart, Aline Freville, Adam J. Reid, Sarah J. Tarr, Kevin K. A. Tetteh, Christian Flueck, Ambroise D. Ahouidi, Alfred Amambua-Ngwa, Mahamadou Diakite, Gordon A. Awandare, David J. Conway

**Affiliations:** a Department of Infection Biology, London School of Hygiene and Tropical Medicine, London, United Kingdom; b Wellcome Sanger Institute, Hinxton, Cambridge, United Kingdom; c Le Dantec Hospital, Université Cheikh Anta Diop, Dakar, Senegal; d MRC Unit The Gambia at London School of Hygiene and Tropical Medicine (LSHTM), Fajara, Banjul, The Gambia; e Malaria Research and Training Center, University of Bamako, Bamako, Mali; f West African Centre for Cell Biology of Infectious Pathogens, Department of Biochemistry, Cell and Molecular Biology, University of Ghana, Legon, Ghana; NIAID/NIH

**Keywords:** antigenic variation, blood culture, malaria, sexual development, transcription

## Abstract

The merozoite surface protein MSPDBL2 of Plasmodium falciparum is under strong balancing selection and is a target of naturally acquired antibodies. Remarkably, MSPDBL2 is expressed in only a minority of mature schizonts of any cultured parasite line, and *mspdbl2* gene transcription increases in response to overexpression of the gametocyte development inducer GDV1, so it is important to understand its natural expression. Here, MSPDBL2 in mature schizonts was analyzed in the first *ex vivo* culture cycle of 96 clinical isolates from 4 populations with various levels of infection endemicity in different West African countries, by immunofluorescence microscopy with antibodies against a conserved region of the protein. In most isolates, less than 1% of mature schizonts were positive for MSPDBL2, but the frequency distribution was highly skewed, as nine isolates had more than 3% schizonts positive and one had 73% positive. To investigate whether the expression of other gene loci correlated with MSPDBL2 expression, whole-transcriptome sequencing was performed on schizont-enriched material from 17 of the isolates with a wide range of proportions of schizonts positive. Transcripts of particular genes were highly significantly positively correlated with MSPDBL2 positivity in schizonts as well as with *mspdbl2* gene transcript levels, showing overrepresentation of genes implicated previously as involved in gametocytogenesis but not including the gametocytogenesis master regulator *ap2-g*. Single-cell transcriptome analysis of a laboratory-adapted clone showed that most individual parasites expressing *mspdbl2* did not express *ap2-g*, consistent with MSPDBL2 marking a developmental subpopulation that is distinct but likely to co-occur alongside sexual commitment.

## INTRODUCTION

The Plasmodium falciparum merozoite surface protein MSPDBL2 is one of two MSP3-like proteins with a duffy-binding like (DBL) domain, expressed in schizonts and colocalized with MSP1 on the merozoite cell surface (1, 2). MSPDBL2 is not directly membrane bound but appears complexed with MSP1 and can bind to the surface of uninfected erythrocytes (1), suggesting a potential role in invasion. The *mspdbl2* gene occurs at a single locus on chromosome 10 with an intact coding sequence in all P. falciparum isolates, although it has multiple stop codons in the related chimpanzee parasite Plasmodium reichenowi, indicating that it is not functional in that species (3, 4) and an orthologue exists only within members of the *Laverania* subgenus but not in other malaria parasites (5). The gene is highly polymorphic within populations where P. falciparum is endemic, and an analysis of allele frequency distributions indicates that diverse alleles are maintained by strong balancing selection (3, 6, 7), suggesting that it may be a target of immune selection.

MSPDBL2 is a target of naturally acquired antibody responses, and population cohort studies where P. falciparum is endemic have shown associations of anti-MSPDBL2 antibodies with a reduced prospective risk of clinical malaria (8, 9), while one study indicated that affinity-purified human anti-MSPDBL2 IgG can inhibit parasites in culture (9). However, the vaccine candidacy of MSPDBL2 is uncertain, as it is not only polymorphic but also highly variable in expression. An analysis of schizont-rich cultures of clinical isolates and long-term adapted P. falciparum laboratory lines has revealed highly variable *mspdbl2* transcript levels, being low in most isolates assayed by reverse transcriptase quantitative PCR (RT-qPCR) (6) or by whole-transcriptome analysis (10). Strikingly, MSPDBL2 protein expression is restricted to a small proportion of mature schizonts in each laboratory-adapted parasite line tested by immunofluorescence with specific antibodies (6). MSPDBL2-positive schizonts coexpress other MSP3-like proteins, including MSPDBL1, MSP3, and MSP6 that are expressed in most or all other schizonts (6). Evaluating transcript levels by RT-qPCR analysis did not indicate a correlation in the expression of the *mspdbl2* gene with any other *msp3-like* gene, indicating that it is independently regulated (6). Consistent with it being expressed in only a small minority of parasites, experimental disruption of the *mspdbl2* gene does not affect overall asexual parasite growth rates in culture (11). However, frequencies of MSPDBL2 expression in schizonts of clinical isolates have not been reported, and its function *in vivo* is unknown.

If MSPDBL2 is restricted to a functionally important parasite subpopulation, it might still be a target to be considered for vaccination. Interestingly, MSPDBL2 has been shown to bind to the Fc region of IgM, although the function of this interaction is unknown (12). Separately, overexpression of *mspdbl2* has been reported to enhance parasite survival in the presence of some antimalarial drugs in culture (13). Significantly, it has been suggested that *mspdbl2* expression may be correlated with parasite sexual differentiation, as overexpression of the *gdv1* gene in an engineered parasite clone results in a marked increase in transcript levels of *mspdbl2* as well as genes known to be involved in switching to gametocyte development (14). This finding indicates the importance of studying variation in *mspdbl2* gene expression as well as MSPDBL2 protein expression in schizonts of clinical isolates and exploring whether this variation associated with markers of parasite commitment to gametocyte development *in vivo*. It is clear that there is great variation in proportions of parasites committed to gametocyte development among different infections from a single area where P. falciparum is endemic (15), and there is correlating variation in levels of transcript markers of ring stage parasites that will develop as gametocytes (16), but apart from the master regulator transcription factor AP2-G (17), there are no known markers of sexually committed schizonts in the previous cycle (18, 19). This study investigates the frequency distribution of expression of the MSPDBL2 protein in schizonts of *ex vivo* cultured clinical isolates from populations where the disease is endemic, revealing that most isolates have very few MSPDBL2-positive schizonts positive while a minority have much higher proportions. A transcriptome sequencing (RNA-seq) analysis of a subset of clinical isolates enables a broad scan for correlations with transcript levels of other genes, indicating a strong excess of genes implicated previously as being associated with the gametocytogenesis pathway. Complementing these findings, a single-cell transcriptome analysis of a laboratory-adapted parasite line is presented, indicating that *mspdbl2* is expressed in different parasites, developing in parallel to those that are sexually committing.

## RESULTS

### Wide variation in proportions of schizonts expressing MSPDBL2 in clinical isolates.

MSPDBL2 protein expression was examined in mature schizonts (each with at least 8 nuclei) developed in the first cultured *ex vivo* cycle of each of 96 P. falciparum clinical isolates sampled from malaria patients in 4 different countries of malaria endemicity in West Africa. A previous analysis of cultured laboratory-adapted P. falciparum lines by immunofluorescence using antibodies against a conserved N-terminal region of MSPDBL2 had shown that individual schizonts were either strongly positive or clearly negative so that mature schizonts could be counted directly to enumerate proportions that were positive (6). To confirm the specificity of the protocol for application in the present study, additional validation was performed by generating a cultured parasite line with MSPDBL2 green fluorescent protein (GFP)-tagged at the C terminus so that double staining could be assessed. The HB3 laboratory-adapted line was modified as it was previously shown to have a higher proportion of schizonts expressing MSPDBL2 than that of other cultured parasite lines (6), and CRISPR-Cas9 genome editing generated the engineered line HB3/MSPDBL2-GFP (see Fig. S1 in the supplemental material). Two-color immunofluorescence microscopy staining confirmed the complete specificity of the anti-MSPDBL2 antibody, as all individual mature schizonts were either clearly positive for both anti-MSPDBL2 and anti-GFP or negative for both (Fig. S1).

10.1128/mbio.01948-22.1 FIG S1 Generation of Plasmodium falciparum line HB3/MSPDBL2-GFP expressing endogenous MSPDBL2 with a GFP tag. (A) Schematic representation of C-terminal GFP tagging of MSPDBL2 in the HB3 strain using the Cas9-mediated genome editing strategy. The position of primers used for diagnostic PCR are indicated by arrows. The recodonized portion of *mspdbl2* is indicated as a blue box. Detection of the 5′ integration was performed with primers P21 (5′-GAAAAGATTTGTGATGTCCT-3′) and P22 (5′-GGAAATATTATTAAAGGAGATGACTTACTCG-3′) (PCR product size, 1,497 bp). Detection of the *mspdbl2* endogenous gene was performed with primers P22 and P24 (5′-CTTCTTCTGTTATATATTCTATTTCCCC-3′) (PCR product size, 1,000 bp). Detection of the tagged version of *mspdbl2* was performed with primers P22 and P23 (5′-GAAACACACAATTAGATTTTTTCCTCAC-3′) (PCR product size, 3,299 bp). (B) Immunofluorescence analysis of the HB3/MSPDBL2-GFP line. Positive schizonts were detected with both a monoclonal mouse anti-GFP (green) and the polyclonal α-MSPDBL2 (red) confirming the specificity of those antibodies, as all parasites were either positive or negative with both antibodies. Parasite nuclei were stained with DAPI (blue). Overall, 9.3% (137/1473) mature schizonts counted expressed MSPDBL2-GFP, a similar proportion to the expression of MSPDBL2 in the original HB3 clone (Amambua-Ngwa *et al.* 2012 PLOS Genetics 8:e1002992). Download FIG S1, TIF file, 0.2 MB. Copyright © 2022 Hocking et al. 2022 Hocking et al. https://creativecommons.org/licenses/by/4.0/ This content is distributed under the terms of the Creative Commons Attribution 4.0 International license.

**FIG 1:**
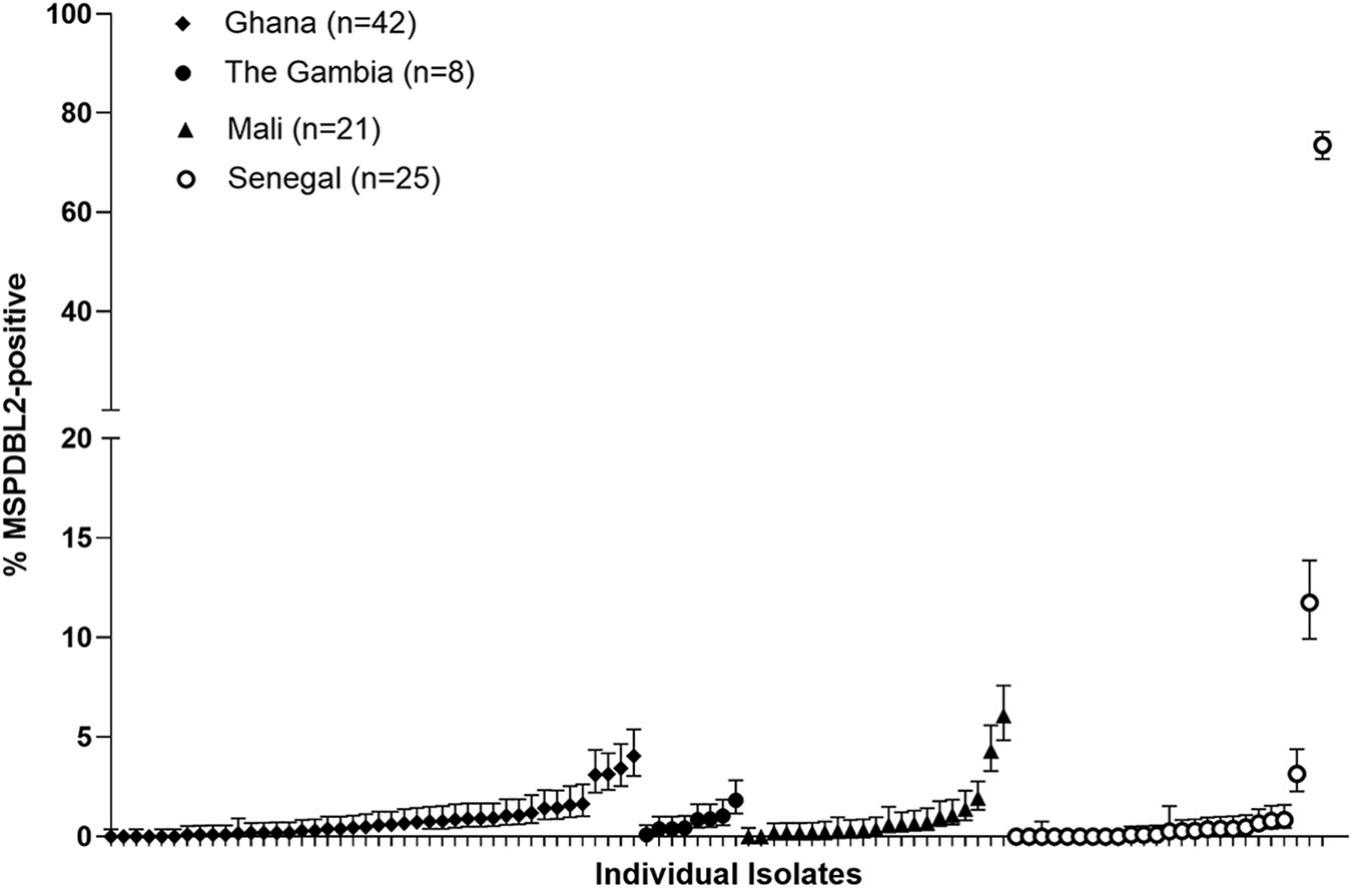
Variable proportions of P. falciparum schizonts expressing MSPDBL2 in *ex vivo* cultures of 96 clinical isolates from four West African countries. Samples were from malaria patients in Ghana (*n* = 42), The Gambia (*n* = 8), Senegal (*n* = 25), and Mali (*n* = 21). In each isolate, approximately 1,000 mature schizonts containing 8 or more nuclei were scored in immunofluorescence assays using polyclonal mouse serum against a conserved N-terminal region of MSPDBL2. For each isolate, the proportions positive and 95% confidence intervals are plotted. Eighteen of the isolates had no schizonts positive, and the median across all isolates was only 0.6%, but proportions were highly skewed and 9 isolates had more than 3% of schizonts positive. Although the two isolates with the highest proportions were from Senegal, there were no significant differences in the overall distributions among different countries (nonparametric overall Kruskall-Wallis test and pairwise Wilcoxon signed-rank test). All numerical data are given fully in Table S1.

In most of the 96 clinical isolates tested in the first cycle of *ex vivo* culture, less than 1% of all mature schizonts were positive for MSPDBL2 by immunofluorescence microscopy (overall median of 0.6%), but the frequency distribution was highly skewed as some isolates had much higher proportions that were positive (Fig. 1; see Table S2 in the supplemental material). Nine isolates had more than 3% schizonts positive, including one that had 73% positive. There were no significant differences in the distributions among different countries (Kruskal-Wallis test and pairwise Mann-Whitney tests were nonsignificant), although the two isolates with highest proportions were from Senegal (Fig. 1). The overall distribution was compared with the data reported previously for a panel of 12 long-term laboratory-adapted P. falciparum lines isolated originally from more diverse sources (6), and the distributions were not significantly different (Mann-Whitney test, *P* = 0.52). This result shows that a wide range of MSPDBL2-positive schizonts, with most isolates having very low positive proportions, is a natural feature of expression in populations where P. falciparum is endemic rather than one which has been selected by laboratory culture.

10.1128/mbio.01948-22.6 TABLE S2 Counts and percentages of MSPDBL2 positivity in mature schizonts (at least 8 nuclei) in *ex vivo* cultures of 96 clinical isolates from 4 populations. Download Table S2, XLSX file, 0.01 MB. Copyright © 2022 Hocking et al. 2022 Hocking et al. https://creativecommons.org/licenses/by/4.0/ This content is distributed under the terms of the Creative Commons Attribution 4.0 International license.

10.1128/mbio.01948-22.7 TABLE S3 RNA-seq details and sequence accession numbers for 17 *P. falciparum* clinical isolates representing a wide range of MSPDBL2 expression in schizonts. Download Table S3, DOCX file, 0.01 MB. Copyright © 2022 Hocking et al. 2022 Hocking et al. https://creativecommons.org/licenses/by/4.0/ This content is distributed under the terms of the Creative Commons Attribution 4.0 International license.

10.1128/mbio.01948-22.8 TABLE S4 List of genes with expression in clinical isolates positively correlating with proportions of schizonts expressing MSPDBL2 protein by IFA at significance *P* values of <0.001. Download Table S4, DOCX file, 0.02 MB. Copyright © 2022 Hocking et al. 2022 Hocking et al. https://creativecommons.org/licenses/by/4.0/ This content is distributed under the terms of the Creative Commons Attribution 4.0 International license.

10.1128/mbio.01948-22.9 TABLE S5 List of genes with expression in clinical isolates negatively correlating with proportions of schizonts expressing MSPDBL2 protein by IFA at significance *P* values of <0.001. Download Table S5, DOCX file, 0.03 MB. Copyright © 2022 Hocking et al. 2022 Hocking et al. https://creativecommons.org/licenses/by/4.0/ This content is distributed under the terms of the Creative Commons Attribution 4.0 International license.

### Transcriptomes of schizont-enriched *ex vivo* cultures with a wide range in proportions of MSPDBL2-positive schizonts.

To explore whether particular parasite gene transcripts are associated with the proportions of MSPDBL2-positive frequencies, whole-transcriptome RNA-seq analysis was performed for 17 of the clinical isolates. The isolates analyzed intentionally included representatives with a wide range of MSPDBL2 expression (0% to 73% of schizonts positive) (see Table S3 in the supplemental material), among those for which sufficient schizont-enriched material was generated in *ex vivo* culture, as noted in Materials and Methods. Sequencing of the cDNA libraries yielded a mean of 2.1 × 10^6^ Illumina short reads for each isolate, and most of these reads aligned to the 3D7 reference genome sequence (Table S3 gives details and sequence accession numbers for each sample), enabling a read depth analysis of relative expression of individual genes after normalization for the total number of reads for each isolate (see Fig. S2 in the supplemental material). The fragments per kilobase per million (FPKM) values across all genes were first compared with published RNA-seq data from tightly synchronized P. falciparum 3D7 parasites sampled at seven time points postinvasion (0, 8, 16, 24, 32, 40, and 48 h, of which the last may include some next cycle reinvasion) (20), confirming that Spearman’s rank correlation expression profiles in all samples had the strongest correlations with schizont stage parasites (13 isolates correlated most strongly with the 40-h time point and 4 isolates with the 32-h time point) (see Fig. S3 in the supplemental material).

10.1128/mbio.01948-22.2 FIG S2 Graphs summarizing variation in sequence read depths among 17 individual clinical isolate cDNA libraries and normalization. (A) The number of genes with zero mapped reads per isolate. (B) The average number of reads per gene. (C) Distribution of the numbers of reads per gene based on raw read data. (D) Variation in the number of reads representing genes is normalized by regularized log (rlog) transformation in DESeq2. Download FIG S2, TIF file, 0.1 MB. Copyright © 2022 Hocking et al. 2022 Hocking et al. https://creativecommons.org/licenses/by/4.0/ This content is distributed under the terms of the Creative Commons Attribution 4.0 International license.

10.1128/mbio.01948-22.3 FIG S3 Correlations of the RNA-seq profiles of each of the 17 clinical isolates with different stages of parasite development. FPKM values for the clinical isolates were correlated with reference data on a laboratory parasite clone across seven timepoints in the P. falciparum intraerythrocytic developmental cycle (Otto *et al.* 2010. *Mol. Microbiol.* 76:12-24). All isolate preparations had their highest correlation with the reference profile at either 32 or 40 h postinvasion. Download FIG S3, TIF file, 0.2 MB. Copyright © 2022 Hocking et al. 2022 Hocking et al. https://creativecommons.org/licenses/by/4.0/ This content is distributed under the terms of the Creative Commons Attribution 4.0 International license.

### Gene transcripts correlating with variable proportions of MSPDBL2-positive schizonts among clinical isolates.

**FIG 2:**
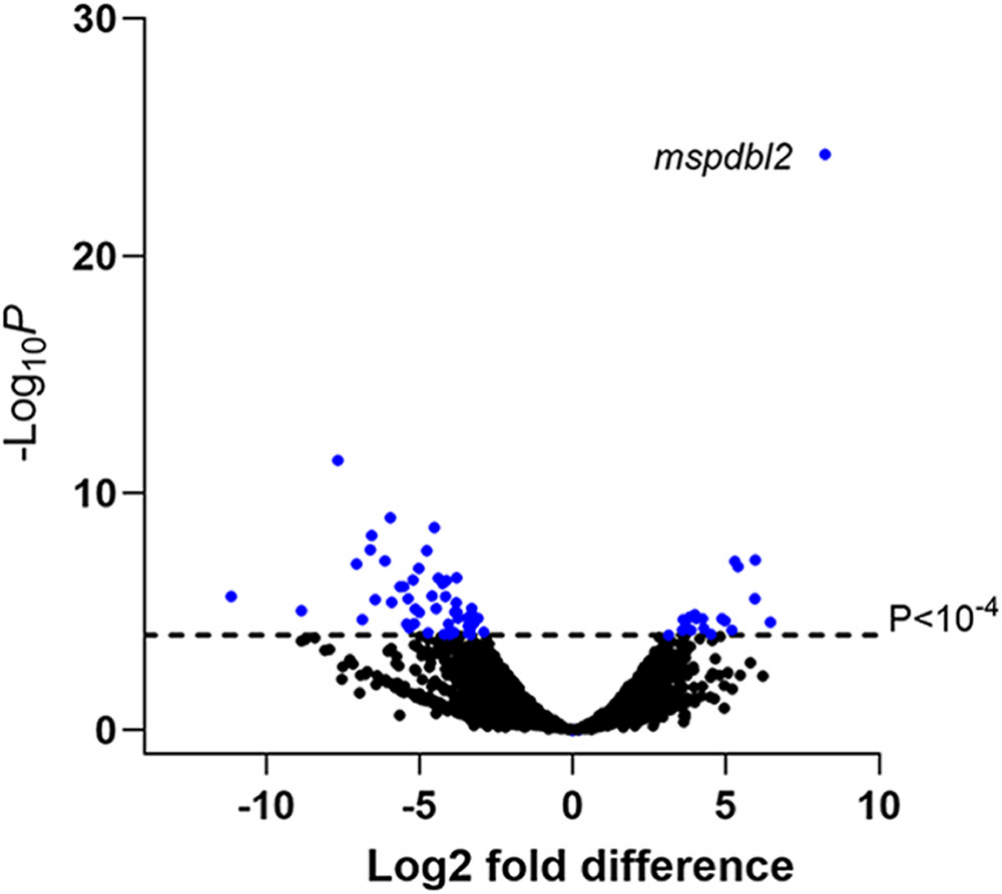
Transcriptome analysis identifies P. falciparum genes showing a correlation with MSPDBL2 expression in *ex vivo*-cultured clinical isolates. RNA-seq was performed on schizont-enriched cultures of 17 West African clinical isolates in the first *ex vivo* cycle, and across the isolates, FPKM transcript levels of each gene were tested for correlation with the proportion of schizonts expressing MSPDBL2. To reduce stochastic noise from low counts, data for isolates with <1% schizonts positive (Table S3) were grouped into three bins (0%, <0.5%, and 0.5 to 0.9% positive). Blue shading indicates those that have the most significant differential gene expression (DGE; *P* < 10^−4^), including the *mspdbl2* gene itself (PF3D7_1036300). The genes positively correlated at this level of significance are listed in Table 1. A broader set of genes correlated positively at a slightly lower level of individual significance (*P* < 0.001) (Table S4), and those that are negatively correlated at that level of significance are also listed separately (Table S5). Fold change estimation was performed by DEseq, with the positive and negative values for all significant genes being shown in Table S4 and S5.

Among the 17 isolates with RNA-seq data, individual gene FPKM relative expression values were tested for correlation with MSPDBL2 indirect fluorescent-antibody assay (IFA) expression (Fig. 2). To scan for significantly correlated genes, a *P* value cut off of <0.001 was initially used which identified 52 genes with increased expression (including the *mspdbl2* gene which was by far the most positively correlated as expected) (see Table S4 in the supplemental material) and 130 genes with negatively correlated expression (see Table S5 in the supplemental material). Cluster analysis of the 17 clinical isolates was performed on the 52 genes that had the most highly significant correlation with proportions of MSPDBL2-positive schizonts, showing diverse clusters of expression across the isolates (see Fig. S4 in the supplemental material). Aside from *mspdbl2* itself, 12 (24%) of the other 51 genes with higher expression were previously listed as having known or suspected roles in gametocytogenesis, whereas of the 130 genes with lower expression, only 10 (7%) were listed with known or suspected gametocytogenesis involvement. An odds ratio of 3.7 (95% confidence interval [CI], 1.5 to 9.2; *P* = 0.0054) on these proportions indicates a significant skew in gametocytogenesis-related genes being more likely to be positively rather than negatively correlated with proportions of MSPDBL2-positive schizonts. The gametocytogenesis master regulator *ap2-g* was not one of the significantly correlating genes.

10.1128/mbio.01948-22.4 FIG S4 Clustering of clinical isolate expression profiles across 52 genes having the highest correlation with proportions of schizonts expressing MSPDBL2 (listed in Table S4). The 17 clinical isolates and gene expression profiles were hierarchically clustered based on pairwise similarity of relative transcript level profiles, with normalized read counts for each gene being extracted and plotted as a heatmap with color shading for each gene. The genes fall into two broad clusters. Asterisks mark the *mspdbl2* gene (Pf3D7_1036300) and 12 genes with known or suspected involvement in gametocytogenesis (among those listed in Table S1). Most of the genes have closer clustering with others than with *mspdbl2* which does not show very close clustering of expression with any particular individual gene. Download FIG S4, TIF file, 0.9 MB. Copyright © 2022 Hocking et al. 2022 Hocking et al. https://creativecommons.org/licenses/by/4.0/ This content is distributed under the terms of the Creative Commons Attribution 4.0 International license.

Using a more stringent correlation value cut off, namely, a *P* value of <10^−4^, to focus on genes having most highly significant correlations with proportions of schizonts expressing MSPDBL2 (Fig. 2), 19 genes are identified as positively correlated, of which 9 (47%) were indicated previously as having known or suspected roles in gametocytogenesis (Table 1). At this level of correlation significance, 51 genes are negatively correlated (Table S3), of which only 1 (2%) was previously indicated as gametocytogenesis related. This finding indicates a very highly significant skew in gametocytogenesis-related genes being positively rather than negatively correlated with proportions of MSPDBL2-positive schizonts, yielding an odds ratio of 45.0 (95% CI, 5.1 to 396.0; *P* = 1.2 × 10^−5^).

**TABLE 1:** P. falciparum genes with most significantly increased expression^*a*^ correlating with proportions of MSPDBL2-positive schizonts in clinical isolates^*b*^

Gene ID^*c*^	*P* value	Gene product description
*PF3D7_1036300*	5.4 × 10^−25^	*duffy binding-like merozoite surface protein 2 (mspdbl2)*
**PF3D7_1476600**	6.7 × 10^−08^	*Plasmodium* exported protein
PF3D7_1474000	7.6 × 10^−08^	Conserved *Plasmodium* protein
**PF3D7_1102500**	1.3 × 10^−07^	GEXP02, *Plasmodium* exported protein (PHISTb)
PF3D7_1461800	3.0 × 10^−06^	Conserved *Plasmodium* protein
PF3D7_1445700	1.4 × 10^−05^	Conserved *Plasmodium* protein
PF3D7_0814200	1.7 × 10^−05^	DNA/RNA-binding protein Alba 1
**PF3D7_1466200**	2.0 × 10^−05^	Early gametocyte enriched phosphoprotein EGXP
**PF3D7_0114000**	2.1 × 10^−05^	GEXP06, exported protein family 1
**PF3D7_1372100**	2.1 × 10^−05^	GEXP04, *Plasmodium* exported protein (PHISTb)
**PF3D7_0215000**	2.2 × 10^−05^	Acyl-CoA synthetase
PF3D7_1362700	2.6 × 10^−05^	Conserved *Plasmodium* protein
**PF3D7_1473700**	2.8 × 10^−05^	Nucleoporin NUP116/NSP116, putative
PF3D7_0829400	2.8 × 10^−05^	Prolyl 4-hydroxylase subunit alpha, putative
PF3D7_1027300	5.2 × 10^−05^	Peroxiredoxin, nuclear protein
PF3D7_0515000	6.2 × 10^−05^	Pre-mRNA-splicing factor CWC2, putative
**PF3D7_1346800**	6.2 × 10^−05^	Pfs47, 6-cysteine protein
PF3D7_1132600	6.4 × 10^−05^	Pre-mRNA-splicing factor 38A, putative
**PF3D7_1477700**	9.1 × 10^−05^	*Pfg14.748, Plasmodium* exported protein (PHISTa)
PF3D7_1431400	9.9 × 10^−05^	Surface-related antigen SRA

a *P* < 10^−4^.

b The most highly correlated transcript is *mspdbl2* itself, as expected. Of the other 19 most significantly correlated genes, 9 (47%) highlighted in bold were implicated previously as potentially gametocytogenesis related (Table S1). Additional genes that positively correlated at the slightly lower significance level of *P* < 0.001, along with the magnitude of the correlations as estimated by DEseq, are listed in Table S4.

c ID, identifier.

10.1128/mbio.01948-22.5 TABLE S1 Reference list of 119 genes considered to be potentially gametocytogenesis elated based on three previous publications (references at the bottom of the table). The list was compiled and fixed prior to the analysis to allow unbiased association testing. Download Table S1, XLSX file, 0.01 MB. Copyright © 2022 Hocking et al. 2022 Hocking et al. https://creativecommons.org/licenses/by/4.0/ This content is distributed under the terms of the Creative Commons Attribution 4.0 International license.

Independently, a previous study investigated the transcriptomic profiles of cultured schizonts of the transgenic P. falciparum parasite line 164/TdTom, comparing preparations enriched for sexually committed versus asexually committed schizonts (21). The data from this study on the PlasmoDB genomics resource site (22) show relative transcript levels accessible for all except 1 of the 19 genes that were most highly positively correlated with MSPDBL2 expression in the present study. Fifteen (83%) of these 18 genes had higher expression in the sexually committed schizont preparation than in the asexual schizont preparation, a significantly positive skew compared with random expectations (*P* < 0.05).

### Genes expressed in correlation with *mspdbl2* gene expression in schizont-enriched cultures of clinical isolates.

To complement the above scan based on proportions of MSPDBL2-positive schizonts, the various transcript levels of *mspdbl2* (FPKM values) among the clinical isolates were analyzed to scan for other genes with correlated expression. Using a correlation significance *P* value of <0.001 identified 41 genes with positively correlated expression (see Table S6 in the supplemental material), of which many were the same as those that also correlated with proportions of MSPDBL2-positive schizonts (Table S4). At a significance *P* value of <0.001, 31 genes had expression negatively correlated with *mspdbl2* (Table S6). Of the 41 genes positively correlated with *mspdbl2* transcript expression, 11 (27%) were previously identified as potentially gametocytogenesis related, compared with only 3 (10%) of the 31 negatively correlated genes, giving an odds ratio of 3.4 (95% CI, 0.9 to 13.6; *P* = 0.06).

10.1128/mbio.01948-22.10 TABLE S6 Two lists of genes with expression in clinical isolates positively or negatively correlating with *mspdbl2* transcript levels at significance *P* values of <0.001. Download Table S6, DOCX file, 0.02 MB. Copyright © 2022 Hocking et al. 2022 Hocking et al. https://creativecommons.org/licenses/by/4.0/ This content is distributed under the terms of the Creative Commons Attribution 4.0 International license.

Using a higher level of cutoff for correlation significance (*P* < 10^−4^), 19 genes were positively correlated with transcript levels of *mspdbl2* (Table 2), of which 8 (42%) were previously identified as potentially gametocytogenesis related, compared with 2 (15%) of 13 genes negatively associated with *mspdbl2*, giving an odds ratio of 4.0 (95% CI, 0.7 to 23.3; *P* = 0.11). In summary, the analysis based on *mspdbl2* transcript levels gives broadly similar results to the analysis based on MSPDBL2-positive schizont proportions, but the excess proportions of positively correlated gametocyte-related genes are less significant.

**TABLE 2:** P. falciparum genes with most significantly increased expression^*a*^ correlating with *mspdbl2* transcript levels measured by FPKM in transcriptomes of clinical isolates

Gene ID^*b*^	*P* value	Gene product description
PF3D7_0114000*	2.6 × 10^−9^	GEXP06, exported protein family 1
PF3D7_1362700*	2.6 × 10^−8^	Conserved *Plasmodium* protein
PF3D7_1466200*	2.2 × 10^−7^	Early gametocyte enriched phosphoprotein EGXP
**PF3D7_1472200**	3.1 × 10^−7^	Histone deacetylase, putative
PF3D7_1467600*	6.0 × 10^−7^	Conserved *Plasmodium* protein
PF3D7_0214300*	1.1 × 10^−6^	Conserved *Plasmodium* protein
PF3D7_1027300*	2.3 × 10^−6^	Peroxiredoxin
PF3D7_1461800*	2.5 × 10^−6^	Conserved *Plasmodium* protein
PF3D7_1473700*	2.9 × 10^−6^	Nucleoporin NUP116/NSP116, putative
PF3D7_1361200*	8.4 × 10^−6^	Conserved *Plasmodium* protein
PF3D7_1474000*	1.0 × 10^−5^	Conserved *Plasmodium* protein
PF3D7_0501400	1.5 × 10^−5^	Interspersed repeat antigen
**PF3D7_0801900**	2.0 × 10^−5^	Lysine-specific histone demethylase, putative
**PF3D7_1408200**	4.9 × 10^−5^	AP2 domain transcription factor AP2-G2
PF3D7_0207800	5.3 × 10^−5^	Serine repeat antigen 3
PF3D7_1235300	7.1 × 10^−5^	CCR4-NOT transcription complex s4, putative
PF3D7_0519500	7.4 × 10^−5^	CCR4 domain-containing protein 1, putative
PF3D7_1228300	7.5 × 10^−5^	NIMA related kinase 1
**PF3D7_1134600**	8.7 × 10^−5^	Zinc finger protein, putative

a *P* < 10^−4^.

b Genes highlighted in bold have known or suspected roles in gametocytogenesis (indicated by prior listing from previous studies) (Table S1). Additional genes that positively correlated at the slightly lower significance level of *P* < 0.001 are listed in Table S6. Asterisks (*) indicate genes that were also identified as having higher expression correlating to MSPDBL2 protein expression in schizonts at significance of *P* < 0.001 (Table S4).

### Single-cell transcriptome analysis of a laboratory-adapted parasite line.

**FIG 3:**
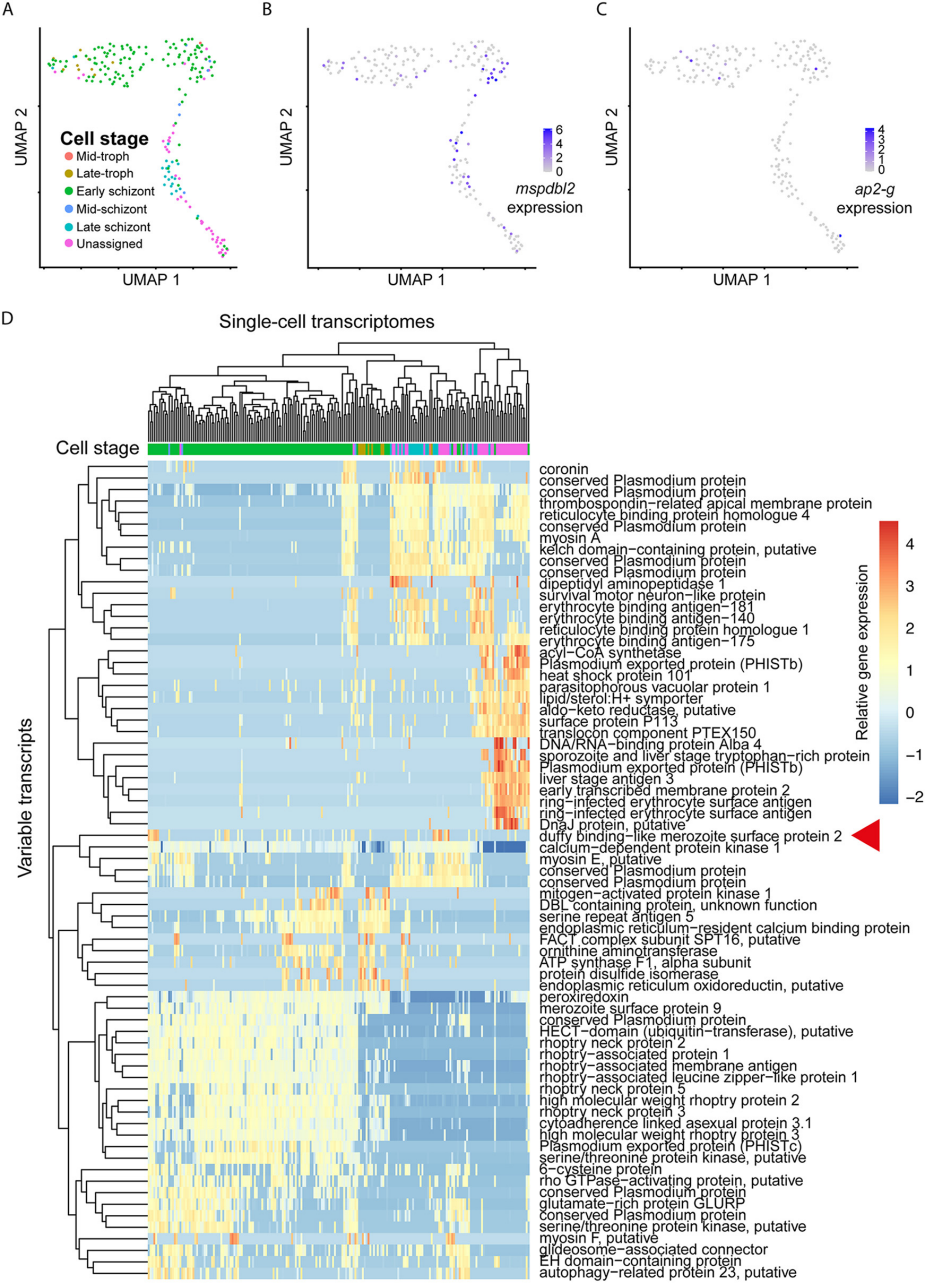
Single-cell RNA-seq analysis of HB3 schizonts. (A) Single-cell transcriptomes of 205 individual HB3 parasites from a schizont-enriched culture are shown in a UMAP dimension reduction plot, with developmental stage estimated by projecting the data onto droplet-based single-cell transcriptomes of P. falciparum 3D7 from the Malaria Cell Atlas. (B) Expression levels of *mspdbl2* plotted on the UMAP embedding of HB3 single-cell transcriptomes, with expression values being scaled by total counts in each cell, multiplied by 10,000, and log transformed. (C) Expression levels of *ap2-g* plotted on the UMAP embedding of HB3 single-cell transcriptomes. (D) Heatmap of variably expressed genes (rows) across the cells (columns). Cells and genes were clustered by hierarchical clustering based on patterns of expression. The *mspdbl2* gene is highlighted with a red triangle.

To test whether *mspdbl2* expression was correlated with levels of expression of *ap2-g* or other genes within individual parasites of a cultured laboratory line, single-cell sequencing was performed on transcriptomes of individual infected erythrocytes sorted from a schizont-enriched culture preparation of the HB3 strain. From the material sorted into 384 wells, cDNA library generation and sequencing were performed, yielding RNA-seq data sufficient for analysis from 205 cells (Fig. 3A). This procedure showed that *mspdbl2* and *ap2-g* were expressed in different cells (Fig. 3B and C), indicating that there is not a direct link between *mspdbl2* expression and sexual commitment. Although many genes had similar expression patterns to others in the data set, no gene showed significant correlation with transcript levels of *mspdbl2* (Fig. 3D). This result indicates the distinctive expression of *mspdbl2*, within separate parasites in parallel to those undergoing sexual commitment.

## DISCUSSION

This study shows that P. falciparum in diverse clinical isolates has a wide range of MSPDBL2 expression positivity in mature schizonts in the first *ex vivo* cycle of development, with most isolates having very low proportions positive. The frequency distribution is similar to that described previously for a smaller number of P. falciparum laboratory-adapted lines that had been cultured for many years (6), indicating that it is not a result of selection by culture adaptation. Furthermore, the frequency distribution was similar in isolates from each of the four populations sampled, which have different levels of malaria infection endemicity within West Africa (23, 24), indicating that parasite populations maintain the wide range of MSPDBL2 expression variation within different endemic environments.

Results of the bulk RNA-seq analyses performed on clinical isolates indicate that MSPDBL2 protein and gene expression in schizonts in the first cycle of development are significantly correlated with the expression of other particular genes within the *ex vivo* cultures. Particularly, many of the most strongly correlated genes were implicated previously as having known or suspected involvement in the process of gametocytogenesis. This result is consistent with a functional study on effects of the gametocyte development gene *gdv1* in assays of an engineered parasite line with highly induced expression of GDV1 (14), which showed significantly increased transcription of *ap2-g* as expected, and also *mspdbl2*, as well as a PHISTa gene (PF3D7_1477700). Another study indicated that levels of the protein encoded by *pfg14_748* increase as parasites develop along the gametocytogenesis pathway, being detectable alongside the early gametocytogenesis marker Pfs16 in parasite cultures before gametocytes were morphologically observed (25). In the present study, the *pfg14_748* gene had expression strongly correlated with *mspdbl2* in the bulk RNA-seq analysis, but although it was shown previously to be induced by GDV1 (14), it is apparently not dependent on the expression of AP2-G (26). Several other genes which correlated with *mspdbl2* expression in the present study (including the nucleoprotein gene *nup116* and the early gametocyte development marker *gexp02*) have been identified as being upregulated by *ap2-g* (26), but *mspdbl2* has not been itself identified to be upregulated by *ap2-g*.

Genes correlating with MSPDBL2 expression in clinical isolates include members of the GEXP family, encoding proteins involved in protein export occurring during gametocytogenesis (27), particularly *gexp02*, *gexp04*, and *gexp06*. As well as *gexp02* being known to result from induced *gdv1* expression (14), it has also been shown that *gexp02* is derepressed in parasites which have conditional knock out of heterochromatin protein 1 (HP1), presumably due to the resulting activation of *gdv1* (28). However, the correlating expression with *gexp02* and other gametocytogenesis genes in this bulk RNA-seq analysis in itself does not indicate that *mspdbl2* is active in the process of sexual commitment. It should be noted that relevant analyses of *gdv1* transcript levels are not possible from the double-stranded cDNA transcriptome data in the present study, as *gdv1* is usually repressed by abundant antisense transcripts initiated from the 3′-intergenic region (14, 29, 30), and ratios of sense to antisense transcripts could be determined only by strand-specific sequencing. Other genes shown previously to be variably expressed in schizont stages of clinical isolates, including different members of the *msp3*-like gene family (6), and members of the *eba* and *Rh* gene families (31, 32), were not among those having a highly significant positive or negative correlation with MSPDBL2 expression here. This finding is consistent with previous results indicating that transcripts of *mspdbl2* in clinical isolates assayed by RT-qPCR were not associated with variation in relative transcript levels of other members of the *msp3*-like family (6) and independent regulation of expression of the other merozoite antigen genes (31, 33, 34).

Other studies have analyzed parasite transcriptomes from earlier stages of development present in peripheral circulation (35–38), although a temporal transcriptome analysis of parasite development through to mature schizonts in the first cycle *ex vivo* has not been performed. The previously published single-cell transcriptome data on P. falciparum schizonts were from a laboratory clone in which hardly any schizonts express MSPDBL2, and therefore, it was not informative on coexpression between *mspdbl2* and other genes (6, 39). Therefore, single-cell RNA-seq was performed here on a P. falciparum clone that was known to have a substantial proportion of schizonts expressing MSPDBL2, which revealed that *mspdbl2* transcripts were not correlated with *ap2-g* transcripts in the same individual parasites. This finding clarifies that the expression of *mspdbl2* occurs in a proportion of parasite schizonts in parallel to those undergoing sexual commitment, both being regulated by *gdv1* (14). This result supports a hypothesis that the MSPDBL2-positive subpopulation has a separate function, contributing to asexual survival and replication under conditions that promote an increased proportion of parasites to undergo terminal sexual differentiation.

## MATERIALS AND METHODS

### P. falciparum clinical isolates.

For an analysis of parasite phenotypic and expression variation in natural infections, clinical malaria cases attending local health facilities in Ghana (Kintampo), Mali (Nioro du Sahel), Senegal (Pikine), and The Gambia (Basse) were investigated. The level of malaria infection endemicity varied among these different populations in West Africa, being higher at the sites in Ghana and Mali than the sites in Senegal and The Gambia, which is also reflected in more complex mixed genome P. falciparum infections at sites where it is more highly endemic, as shown previously for these populations (23). Patients aged between 2 and 14 years were eligible if they had uncomplicated clinical malaria, had not taken antimalarial drugs in the 72 h preceding sample collection, and tested positive for P. falciparum malaria by lateral flow rapid diagnostic test and slide microscopy. Written informed consent was obtained from parents or legal guardians of participating children, and additional assent was received from participating children. Up to 5 mL of venous blood was collected in heparinized anticoagulation BD Vacutainer tubes (BD Biosciences), and a proportion of the erythrocytes were cryopreserved in glycerolyte and stored at −80°C or in liquid nitrogen before shipment on dry ice to the London School of Hygiene and Tropical Medicine for subsequent culture and laboratory analysis. Ethical approval for the collection and analysis of clinical samples was granted by the ethics committees of the Ministry of Health in Senegal, the Ministry of Health in Mali, the Ghana Health Service, the Noguchi Memorial Institute for Medical Research, University of Ghana, Kintampo Health Research Centre, Medical Research Council (MRC) Gambia, and the London School of Hygiene and Tropical Medicine.

### P. falciparum schizont preparations from the first cycle of *ex vivo* culture.

The clinical blood samples were thawed in batches of eight at a time and introduced into culture *ex vivo*, with all isolates being processed in culture in a single laboratory, as described for a previous study of the first *ex vivo* malaria parasite generation from similar clinical samples (31). Giemsa-stained thin blood films were prepared for each isolate initially upon thawing, and later during the second day of culture to assess the developmental progression of parasites into schizogony. Isolates containing schizonts in culture on the second day after thawing were enriched for schizonts by magnetic magnetic-activated cell sorting (MACS) separation, and parasites were then allowed to mature in the presence of E64 for 4 h to prevent schizont rupture, following which cells were harvested by centrifugation, using methods similar to those applied previously to studies of schizonts in continuously cultured parasite lines (10). Erythrocytes containing matured schizonts were prepared for immunofluorescence assays by washing and resuspending in 1% bovine serum albumin (BSA) and spotting into individual wells of 12-well slides (Hendley-Essex), air dried, and stored with desiccant at −40°C until they were assayed, as performed in a previous analysis of schizonts in cultured parasite lines (6).

### GFP tagging of endogenous MSPDBL2 in a control parasite line.

A genetically engineered P. falciparum line, HB3/MSPDBL2-GFP, was produced by modifying the endogenous *mspdbl2* gene locus in the laboratory-adapted parasite clone HB3 so that the protein was tagged with green fluorescent protein (GFP) at the C-terminal end, using the Cas9-mediated genome editing strategy (40). The repair plasmid pGEM3ZF-EGFP was first modified from a pGEM3ZF plasmid (Promega) to contain a GFP-encoding sequence (using primers P13, 5′-CGGTACCCGGGGATCGGATCCAGTAAAGGAGAAGAACTTTTCACT-3′; and P14, 5′-GCAGGTCGACTCTAGTCTAGATTATTTGTATAGTTCATCCATGCCATGTG-3′). A portion of the *mspdbl2* gene (named HR1) comprising a homologous region (nucleotide positions 840 to 1491) and a recodonized region (nucleotide positions 1492 to 2295) without the stop codon was synthetized commercially (Integrated DNA Technologies) and inserted by fusion cloning into pGEM3ZF-EGFP in phase with the e*gfp* 5′ end. Subsequently, a portion of the 3′ untranslated region (UTR) region (referred to as HR2, 1 kb after the stop codon) was inserted fused to the 3′ end of e*gfp* (using primers P15, 5′-AGACTAGAGTCGACCCTGCAGTAATAATTAAAAGGTAAATAAATAAATATAAATATAAA-3′; and P16, 5′-GAATACTCAAGCTTGGCATGCAGGAAATATTATATTTTACATTTTTATGTAATTGCTTAATTTAC-3′). Single guide RNA (sgRNA)-containing plasmids were generated from a pDC2-based plasmid (41) by replacement of the BtgZI adaptor with an sgRNA sequence. The following two plasmids were generated: sgRNA-A (5′-CCAAATACAGATGATAACAGTGG-3′) and sgRNA-B (5′-ACACAACAGGAAAATCAACCTGG-3′). Primers corresponding to those sequences were annealed and inserted into the BtgZI-digested plasmid using the In-Fusion HD cloning strategy (Takara Bio USA, Inc.) as described (40). The repair plasmid was cotransfected in the HB3 parasite line with both sgRNA-containing plasmids, and the transfected culture was treated for 3 days with the antifolate drug WR 99210. Successful integration was analyzed by PCR (using primers specified in Fig. S1), and schizonts were analyzed by immunofluorescence microscopy with a polyclonal rabbit anti-GFP primary antibody (Invitrogen; A11122) at 1/500 dilution and Alexa Fluor 488-conjugated anti-rabbit IgG (Invitrogen; A3273) as the secondary antibody at 1/1,000 dilution. The specificity of the anti-MSPDBL2 murine polyclonal antibody was confirmed by immunofluorescence on this modified parasite line, with Alexa Fluor 594-conjugated anti-mouse IgG (Invitrogen; A11032) as the secondary antibody at 1/1,000 dilution, double staining (in red) the schizonts that were also GFP-positive. Parasite imaging was performed on a EVOS FL (Life Technologies) fluorescence microscope.

### Analysis of MSPDBL2 expression in schizonts by immunofluorescence.

Staining of schizonts positive for MSPDBL2 by immunofluorescence was performed with the same method used to analyze laboratory-adapted P. falciparum isolates in a previous study (6). Briefly, multiwell slides of parasite cultures were air dried and stored under desiccation at −40°C until required. Prior to the assay, fixation was performed for 30 min with 4% paraformaldehyde and 0.0075% glutaraldehyde, before washing for 10 min in 0.1% Triton X-100 and blocking overnight with 3% BSA in PBS. Wells were incubated with a 1/500 dilution of polyclonal mouse serum specific for the conserved N-terminal portion of MSPDBL2 and subsequently with goat anti-mouse IgG Alexa Fluor 555 secondary antibody, using Vectashield mounting fluid containing 4′,6-diamidino-2-phenylindole (DAPI) to visualize parasite nuclei. This method has previously enabled a straightforward and consistent scoring of the proportions of mature schizonts expressing MPDBL2 and shows virtually all schizonts as positive when using antibodies against commonly expressed antigens (8). For each isolate, approximately 1,000 mature schizonts (each containing at least 8 nuclei) were counted using DAPI and individually scored for MSPDBL2 expression using a manual Leica fluorescence microscope with a 100× objective. MSPDBL2 expression was always clearly brightly positive or entirely negative in each individual mature schizont, so those counts of numbers and proportions positive in each preparation were recorded, using the same process as described previously (6). In the present study, the precision of the method was further validated by an analysis of a new parasite line with GFP-tagged MSPDBL2, to check that it was the same individual schizonts expressing GFP that also stained with the anti-MSPDBL2 antibodies.

### RNA-seq of schizont-enriched samples of clinical isolates.

Parasite schizont-enriched *ex vivo* culture material from individual isolates was stored in either TRIzol or RNA*later* (Thermo Fisher Scientific, MA), and RNA was extracted by phenol-chloroform and cleaned up using the NucleoSpin RNA XS extraction kit (Macherey-Nagel, Germany). As the MSPDBL2 protein is specifically expressed in schizonts and as the *ex vivo* cultures contained limited material that precluded multiple time point sampling, the RNA-seq analysis was applied to these preparations for which sufficient RNA was obtained. Samples showing successful RNA extraction after checking by Bioanalyzer electrophoresis were reverse transcribed, and cDNA was amplified using the SmartSeq v4 ultra-low input RNA kit for sequencing (TaKaRa Bio. Inc., Shiga Prefecture, Japan). Successfully amplified samples were prepared for paired-end short-read sequencing using the Nextera XT library prep kit (Illumina, CA), with individual libraries being pooled in equimolar amounts at 4 nM with up to 12 per pool, and sequencing was performed on an Illumina MiSeq instrument using the 150-cycle MiSeq reagent kit v3. RNA from isolate INV236 which had the highest proportion of MSPDBL2-positive schizonts (73%) was prepared and sequenced as a priority, and following this step, RNA was extracted from 38 other isolates with various proportions of MSPDBL2-positive schizonts, of which 25 showed the expected cDNA size range profile after reverse transcription and amplification, and 16 of themwere selected for sequencing as they had an RNA quality RNA integrity number (RIN) score of >6. This procedure yielded a set of 17 isolates with RNA-seq data and matched IFA data on proportions of MSPDBL2-positive schizonts.

Following procedures used previously for the RNA-seq analysis of schizont-enriched P. falciparum cultures of other isolates (10), whole-transcriptome short-read sequence data were assembled by alignment mapping to the P. falciparum 3D7 v3.0 reference genome (42) using HISAT2 (43). Gene transcript levels were assessed using the fragments per kilobase of transcript per million mapped reads (FPKM) metric (the number of reads mapping to each gene normalized for the size of the sequencing library and for gene length). Data were analyzed using the R package DESeq2 (44), using a masked GFF annotation file that removed the *var*, *rifin*, and *stevor* gene families from analysis, as described for the analysis of previous schizont transcriptome data (10). In addition, portions of other protein-coding genes that show high allelic diversity (including the highly polymorphic central region of the *mspdbl2* gene) were masked to ensure mapping occurred only in conserved regions of those genes, as described previously (10). Prior to the analysis, three independent studies, including proteomic or transcriptomic analyses, were consulted (14, 26, 27) to identify P. falciparum genes considered to be potentially associated with gametocytogenesis, enabling the compilation of a list of 119 genes (see Table S1 in the supplemental material) used as a set for conducting exploratory correlative analyses of the RNA-seq data within this study. To avoid discovery bias in the present study, it needed to be fixed prior to analysis but is not a reference list for future studies, as ongoing research means that any such compilations should be updated and can be subject to different criteria.

### Single-cell transcriptome analysis of HB3 strain parasite schizonts.

A schizont-enriched preparation of the P. falciparum line HB3, which consistently has a higher proportion of schizonts expressing MSPDBL2 than other laboratory-adapted lines (6), was flow sorted into individual wells of a 384-well plate so that each well should contain a single infected erythrocyte. Erythrocytes infected with parasites containing hemozoin were purified using LD magnetic columns (Miltenyi Biotec) and incubated with the cysteine protease inhibitor E64 (Thermo Fisher) for 5 h, following which the cells were centrifuged at 200 × *g* for 5 min with a slow brake. The supernatant was removed, and the remaining cells were resuspended in sorting medium (Sigma P7509 phenol red-free RPMI with 0.5% Albumax 0.22 μm filtered, 0.1 mM hypoxanthine, and 25 mM HEPES [pH 7.3]) before being passed through a 40-μm cell strainer. Resuspended cells were diluted to yield a cell density of 1 × 10^7^ mL^−1^, and the material was split into 2 tubes, with Vybrant DyeCycle green stain being added to one tube while the other remained unstained. Uninfected red blood cells were also prepared for sorting and stained as above. Cells were sorted using a FACs Aria Fusion cell sorter, with uninfected red blood cells being first processed through the cell sorter to set gates for debris and red blood cells. Singlets were sorted using forward scatter (FSC) versus side scatter (SSC). A total of 10,000 events from the Vybrant DyeCycle green-stained cells (488 nm, filter 530/30 fluorescein isothiocyanate [FITC]) were sorted to gate on DNA-positive parasite-infected cells. Using this gating, unstained cells were sorted into individual wells which each contained 2 μL of lysis buffer (NEB cell lysis buffer, incorporating RNase inhibitor murine E6429B and nuclease-free water).

After cells were sorted, the plate was frozen and stored at −80°C, prior to RNA-seq library preparation from each of the individual wells using the NEBNext single-cell/low input RNA library prep kit for Illumina with 26 cycles of PCR at the cDNA stage. The individual libraries were sequenced on a single Illumina HiSeq 4000 lane with 75-bp paired-end reads. Reads were demultiplexed and Nextera adaptor sequences were trimmed using *trim_galore -q 20 -a* *CTGTCTCTTATACACATCT* *–paired –stringency 3 –length 50 -e 0.1* (v0.6.4). HISAT2 (v2.0.0-beta) (43) indexes were produced for the P. falciparum v3 genome sequence and annotation (42). Trimmed, paired reads were mapped to either genome sequence using *hisat2 –max-intronlen 5000 p 12*. Reads were summed against genes using HTSeq as follows: *htseq-count -f bam -r pos -s no –t CDS* (v0.11.2) (45). HTSeq excludes multimapping reads by default (-a 10), which means that reads mapping ambiguously to similar genes from the same family are not considered in our analysis. Read counts were processed using Seurat v4.0.2 (46). Transcripts detected in fewer than 3 cells were excluded, and cells with fewer than 100 transcripts detected were excluded. Reads were normalized using the *LogNormalize* function, with scale factor of 10,000. Data were scaled using all genes. Uniform manifold approximation and projection (UMAP) plots were drawn using Seurat, with default parameters.

To determine the cell stage of each transcriptome, the HB3 strain single-cell transcriptomes were projected onto those of droplet-based (10× Chromium) transcriptomes of the 3D7 strain from the Malaria Cell Atlas (39). They provide a description of the P. falciparum intraerythrocytic development cycle and are labeled with cluster annotations, as follows: early-ring, late-ring, early-troph, midtroph, late-troph, early-schizont, midschizont, and late-schizont. The HB3 Seurat object was converted to a single-cell experiment object using Package *SingleCellExperiment* v1.12.0 (47). SCMAP v1.12.0 (48) was then used to project the HB3 data onto the Malaria Cell Atlas 3D7 transcriptomes. To examine whether *mspdbl2* had a similar expression pattern to other genes, we identified those genes which varied in expression across the data set using M3Drop v1.16.0 (49) (mt_method = FDR, mt_threshold = 0.01).

### Statistical analyses.

Tests for the significance of correlations between different variables (including proportions of schizonts positive for MSPDBL2 and individual gene FPKM values) or estimations of odds ratios and significance of associations between categorical variables were conducted using a combination of R, Epi-Info, and Prism software. Differential gene expression analysis was carried out in DESeq2 (44), testing for significant differences and correlations in individual gene expression profiles based on the negative binomial distribution, based on the distributions of derived FPKM values for each gene as defined above.

### Data availability.

The bulk RNA-seq transcriptome data for the 17 clinical isolates have been made available on the NCBI Gene Expression Omnibus (GEO) with accession numbers GSM5897922 to GSM5897938. The single-cell transcriptome data set for HB3 has been made available in the European Nucleotide Archive accession ERP116861.
