# Supplementary figures and images for: Highly Variable Expression of Merozoite Surface Protein MSPDBL2 in Diverse Plasmodium falciparum Clinical Isolates and Transcriptome Scans for Correlating Genes
Source: mBio. 2022 Aug 11;13(4):e01948-22. doi: 10.1128/mbio.01948-22 (PMC9426457; doi:10.1128/mbio.01948-22)

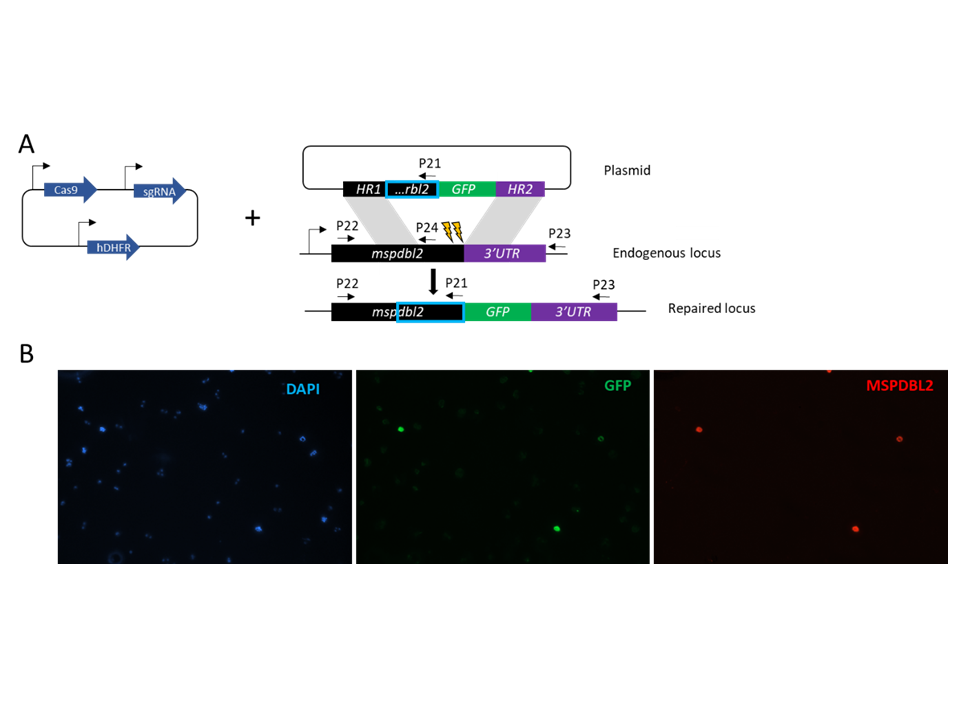

Supplement: FIG S1 [file mbio.01948-22-s0001.tif]

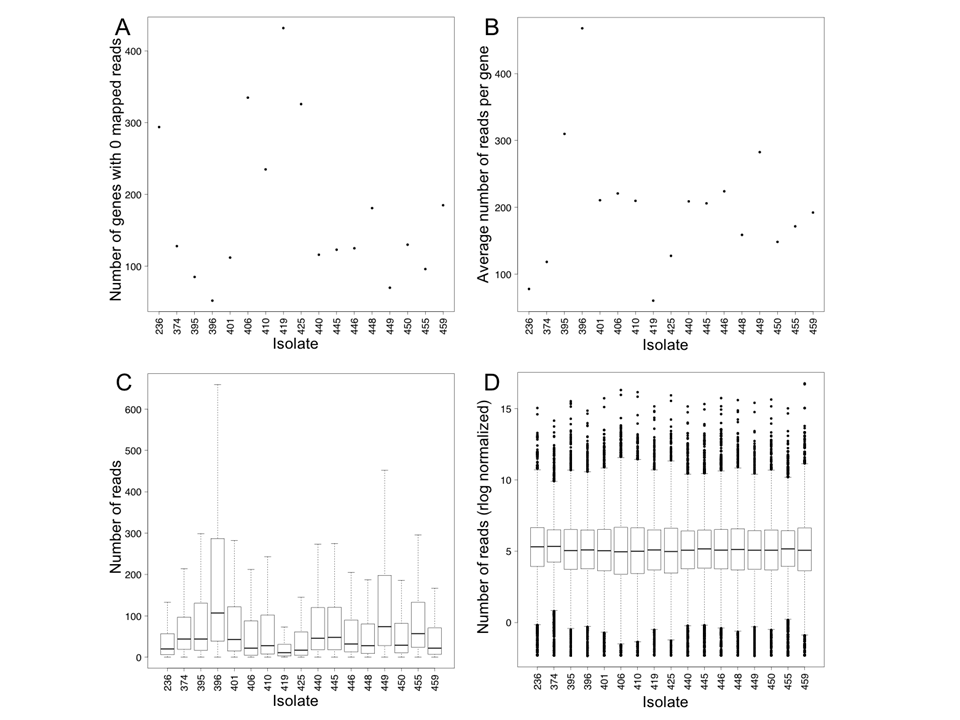

Supplement: FIG S2 [file mbio.01948-22-s0006.tif]

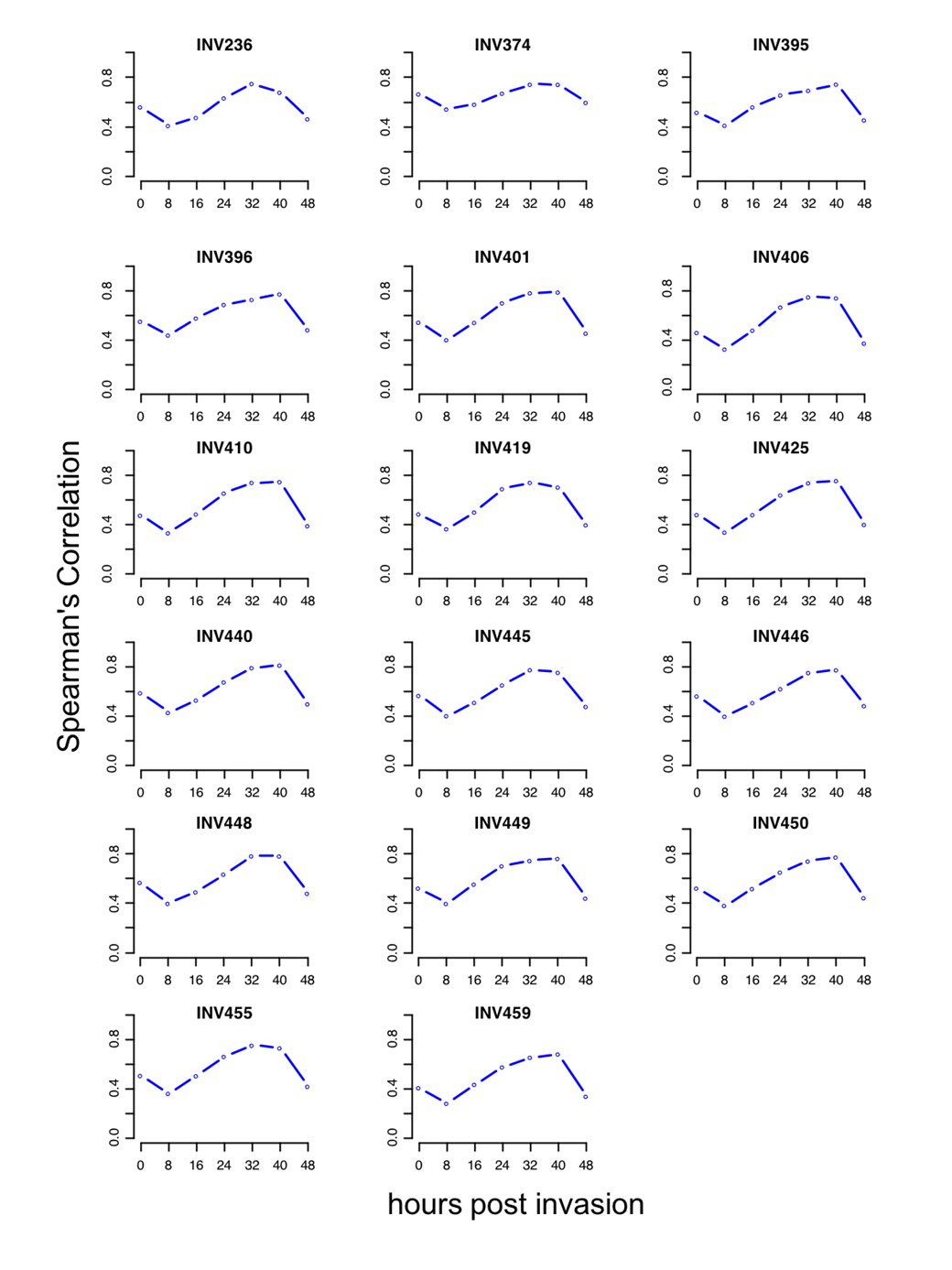

Supplement: FIG S3 [file mbio.01948-22-s0007.tif]

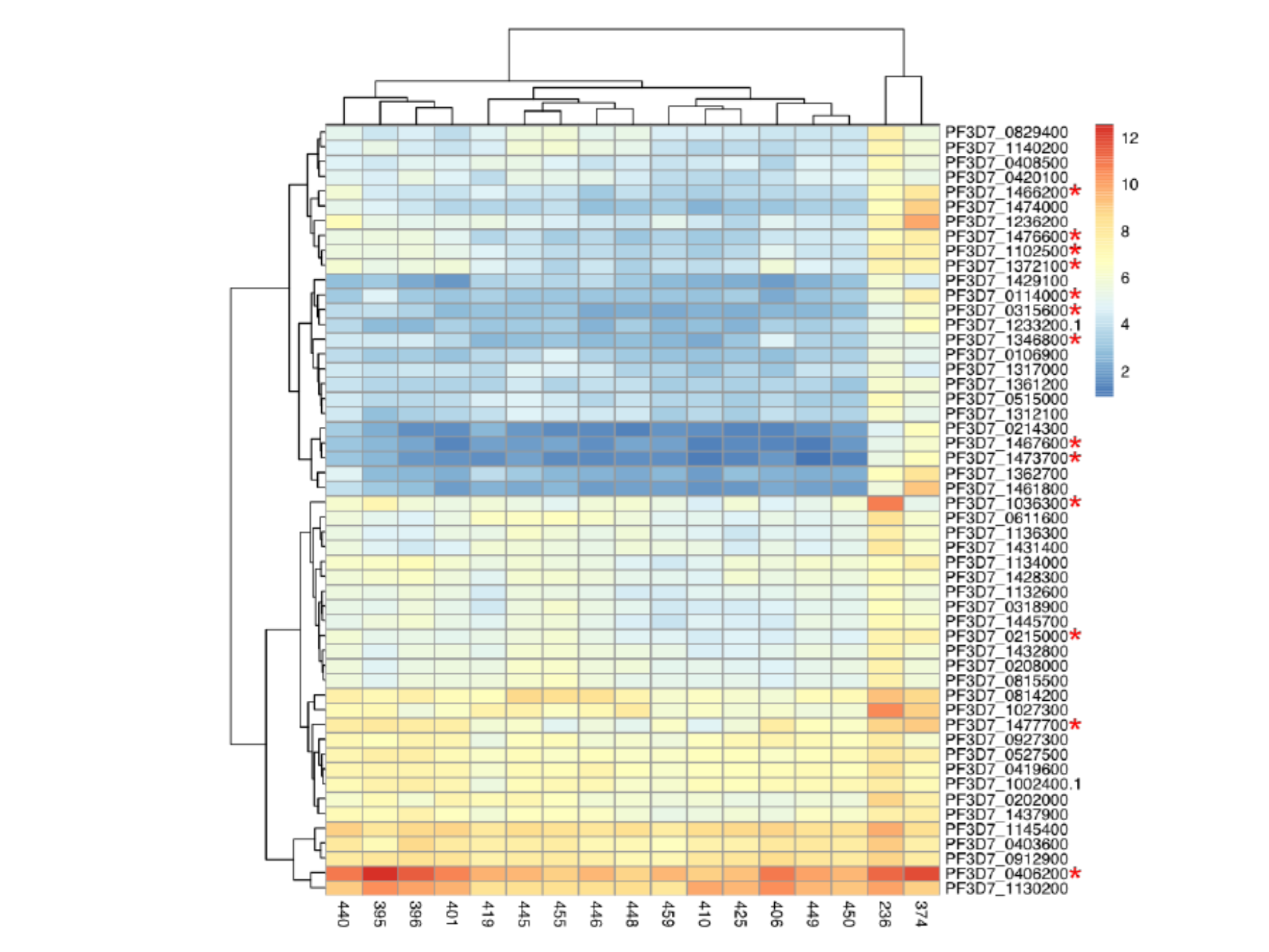

Supplement: FIG S4 [file mbio.01948-22-s0008.tif]
