# Supplementary material for: Highly Variable Expression of Merozoite Surface Protein MSPDBL2 in Diverse Plasmodium falciparum Clinical Isolates and Transcriptome Scans for Correlating Genes
Source: mBio. 2022 Aug 11;13(4):e01948-22. doi: 10.1128/mbio.01948-22 (PMC9426457; doi:10.1128/mbio.01948-22)
Supplement: TABLE S6 [file mbio.01948-22-s0010.docx]

**Supplementary Table S6. Two lists of genes with expression in clinical isolates positively or negatively correlating with *mspdbl2* transcript levels at significance values of P < 0.001**

**S6A. List of 41 genes with expression in clinical isolates positively correlating with *mspdbl2* transcript levels measured by FPKM at significance values of P < 0.001**

| **Gene ID** | **P-value** | | **Product Description** |
| --- | --- | --- | --- |
| **PF3D7_0114000*** | 2.63 E-09 | exported protein family 1 | |
| PF3D7_1362700* | 2.60 E-08 | conserved *Plasmodium* protein, UF | |
| **PF3D7_1466200*** | 2.17 E-07 | early gametocyte enriched phosphoprotein EGXP | |
| **PF3D7_1472200** | 3.09 E-07 | histone deacetylase, putative | |
| **PF3D7_1467600*** | 6.00 E-07 | conserved *Plasmodium* protein, UF | |
| PF3D7_0214300* | 1.11 E-06 | conserved *Plasmodium* protein UF | |
| PF3D7_1027300* | 2.30 E-06 | Peroxiredoxin | |
| PF3D7_1461800* | 2.52 E-06 | conserved *Plasmodium* protein, UF | |
| **PF3D7_1473700*** | 2.89 E-06 | nucleoporin NUP116/NSP116, put | |
| PF3D7_1361200* | 8.35 E-06 | conserved *Plasmodium* protein, UF | |
| PF3D7_1474000* | 1.01 E-05 | conserved *Plasmodium* protein, UF | |
| PF3D7_0501400 | 1.49 E-05 | interspersed repeat antigen | |
| **PF3D7_0801900** | 2.01 E-05 | lysine-specific histone demethylase, put | |
| **PF3D7_1408200** | 4.85 E-05 | AP2 domain transcription factor AP2-G2 | |
| PF3D7_0207800 | 5.33 E-05 | serine repeat antigen 3 | |
| PF3D7_1235300 | 7.10 E-05 | CCR4-NOT transcription complex s4, put | |
| PF3D7_0519500 | 7.43 E-05 | CCR4 domain-containing protein 1, put | |
| PF3D7_1228300 | 7.52 E-05 | NIMA related kinase 1 | |
| **PF3D7_1134600** | 8.65 E-05 | zinc finger protein, putative | |
| **PF3D7_0315600*** | 1.81 E-04 | zinc finger protein, putative | |
| PF3D7_1133700 | 1.27 E-04 | FHA domain-containing protein, put | |
| PF3D7_1236200* | 1.33 E-04 | conserved *Plasmodium* protein, UF | |
| PF3D7_1212700 | 1.49 E-04 | eukaryotic translation initiation factor 3.A, putative | |
| PF3D7_1233200* | 1.57 E-04 | conserved *Plasmodium* protein, UF | |
| PF3D7_1327300 | 2.35 E-04 | conserved *Plasmodium* protein, UF | |
| **PF3D7_1102500*** | 2.40 E-04 | *Plasmodium* exported protein (PHISTb), UF | |
| PF3D7_1469600 | 2.51 E-04 | acetyl-CoA carboxylase | |
| PF3D7_0309200 | 3.31 E-04 | serine/threonine protein kinase, putative | |
| PF3D7_0724100 | 3.94 E-04 | conserved *Plasmodium* protein, UF | |
| PF3D7_0723400 | 4.46 E-04 | conserved *Plasmodium* protein, UF | |
| PF3D7_0829400* | 5.28 E-04 | prolyl 4-hydroxylase subunit alpha, putative | |
| PF3D7_1132400 | 5.86 E-04 | conserved *Plasmodium* membrane protein, UF | |
| PF3D7_1138800 | 6.24 E-04 | WD repeat-containing protein, putative | |
| PF3D7_1142100 | 7.22 E-04 | conserved *Plasmodium* protein, UF | |
| PF3D7_1014300 | 7.43 E-04 | SPRY domain-containing protein, putative | |
| PF3D7_1437200 | 8.05 E-04 | ribonucleoside-diphosphate reductase subunit | |
| PF3D7_0930300 | 9.74 E-04 | merozoite surface protein 1 | |
| PF3D7_1133800 | 9.79 E-04 | RNA (uracil-5-)methyltransferase, putative | |
| **PF3D7_1148700** | 9.80 E-04 | *Plasmodium* exported protein (PHISTc), UF | |
| PF3D7_0402200 | 9.99 E-04 | surface-associated interspersed protein 4.1 pseudo | |

Genes highlighted bold have known or suspected roles in gametocytogenesis. Asterisks * indicate genes also identified as having higher expression correlating to MSPDBL2 protein expression in schizonts by IFA. UF: protein has unknown function.

**S6B. List of 31 genes with expression in clinical isolates negatively correlating with *mspdbl2* transcript levels measured by FPKM at significance values of P < 0.001**

| Gene ID | P-value | Product Description |
| --- | --- | --- |
| PF3D7_0805200 | 2.04E-08 | gamete release protein, putative |
| PF3D7_1416500 | 4.01E-07 | NADP-specific glutamate dehydrogenase |
| PF3D7_1102700 | 7.05E-07 | early transcribed membrane protein 11.1 |
| PF3D7_0202500 | 2.08E-06 | early transcribed membrane protein 2 |
| PF3D7_0402100 | 1.15E-05 | Plasmodium exported protein (PHISTb), unknown function |
| PF3D7_0605900 | 1.20E-05 | long chain polyunsaturated fatty acid elongation enzyme |
| PF3D7_1401400 | 1.74E-05 | early transcribed membrane protein 14.1 |
| PF3D7_1337800 | 2.15E-05 | calcium-dependent protein kinase 5 |
| PF3D7_0424600 | 2.29E-05 | Plasmodium exported protein (PHISTb), unknown function |
| PF3D7_1238900 | 5.77E-05 | protein kinase 2 |
| PF3D7_1240100 | 7.69E-05 | early transcribed membrane protein 12 |
| PF3D7_1324400 | 8.22E-05 | PRELI domain-containing protein, putative |
| PF3D7_1102800 | 9.96E-05 | early transcribed membrane protein 11.2 |
| PF3D7_0702200 | 1.05E-04 | lysophospholipase, putative |
| PF3D7_1218500 | 1.54E-04 | dynamin-like protein, putative |
| PF3D7_0210000 | 1.81E-04 | secretory complex protein 61 gamma subunit |
| PF3D7_1477800 | 1.96E-04 | acyl-CoA binding protein |
| PF3D7_1401100 | 2.16E-04 | DnaJ protein, putative |
| PF3D7_1476800 | 2.26E-04 | lysophospholipase, putative |
| PF3D7_1404800 | 2.50E-04 | conserved Plasmodium protein, unknown function |
| PF3D7_1102400 | 2.69E-04 | phosphopantothenate--cysteine ligase, putative |
| PF3D7_0916700 | 2.97E-04 | RNA-binding protein musashi, putative |
| PF3D7_0112900 | 3.58E-04 | Plasmodium exported protein, unknown function |
| PF3D7_1472600 | 5.08E-04 | protein disulfide-isomerase |
| PF3D7_0730800 | 5.26E-04 | Plasmodium exported protein, unknown function |
| PF3D7_0206200 | 5.56E-04 | pantothenate transporter |
| PF3D7_1253100 | 5.87E-04 | Plasmodium exported protein (PHISTa), unknown function |
| PF3D7_1245000 | 6.15E-04 | 5-formyltetrahydrofolate cyclo-ligase, putative |
| PF3D7_1312500 | 6.79E-04 | conserved Plasmodium protein, unknown function |
| PF3D7_1104100 | 7.47E-04 | syntaxin, Qa-SNARE family |
| PF3D7_1201200 | 8.95E-04 | Plasmodium exported protein (PHISTa-like) |
